# The use of the “Lansaka Model” as the larval indices surveillance system for a sustainable solution to the dengue problem in southern Thailand

**DOI:** 10.1371/journal.pone.0201107

**Published:** 2018-08-01

**Authors:** Charuai Suwanbamrung, Chanchuri Thoutong, Thidarat Eksirinimit, Supapon Tongjan, Kanapot Thongkew

**Affiliations:** 1 School of Public Health, Walailak University, Nakhon Si Thammarat, Thailand; 2 School of Nursing, Walailak University, Nakhon Si Thammarat, Thailand; 3 Center for Computer Services, Walailak University, Nakhon Si Thammarat, Thailand; 4 Center of Department Control 11, Nakhon Si Thammarat, Thailand; The University of Warwick, UNITED KINGDOM

## Abstract

Background: Dengue has been spreading in Thailand for more than 50 years, and the community prevention of dengue transmission is an important strategy to help reduce outbreaks. The larval indices surveillance system is one of the most significant prevention methods at the household and district levels. Objective: This study sought to develop a larval indices surveillance system based on a specific community context. Method: Community participation action research (CPAR) studies represent a new approach to studying the high-risk dengue area of Lansaka district, Nakhon Si Thammarat province, Thailand. This study was conducted for 2 years (from 2013 to 2015) and applied the integrated concepts of 1) community capacity building, 2) epidemiology, 3) research design for health development, and 4) an online computer program. The method included five phases: 1) community preparation, 2) situation assessment, 3) the development of the surveillance system, 4) implementation, and 5) evaluation. Results: The model was designed in partnership with all the stakeholders from 44 villages across 5 sub-districts. The surveillance system consisted of seven steps at the household level based on primary care surveillance centers (PCSCs), as well as four components at the district level based on district surveillance centers (DSCs). The dengue morbidity rate decreased from 164, 151, and 135 cases/100,000 people in 2014, 2015, and 2016, respectively. Moreover, knowledge of both dengue and larval indices among village health volunteers (VHVs) increased significantly (*p* < .01). Conclusions: The results from the new system showed a decrease in both the larval indices level and morbidity rate; however, the levels remained higher than the standard. The active surveillance system requires continuous monitoring at both the household and district levels.

## Introduction

Dengue has become a significant health problem for several countries around the world. An estimated 2.5 billion people are at risk of infection, including approximately 975 million who live in tropical and sub-tropical countries [1]. Dengue has also been a significant health problem in Thailand; in the southern part of the country, both a high morbidity rate and high larval indices are present. A higher dengue incidence has been detected in this area compared with other areas of Thailand [2]. In particular, the southern region of Nakhon Si Thammarat province is at a high risk of dengue fever compared with other provinces in this region because of several factors, including rainfall, temperature [1,3], population density, types of dengue [4,5], non-specific treatment, lack of a successful vaccine [6], ineffective drugs [7], and attitudes toward dengue prevention [8,9]. Dengue transmission in the community is one important consideration [5, 10, 11] for dengue prevention. However, this method requires community participation and community capacity [12, 13] to decrease the larval indices level. Larval index surveys such as the House Index (HI, which measures the percentage of houses infested with larvae), the Breateau index (BI, which measures the number of positive containers per 100 houses inspected), the Container Index (CI, which measures the percentage of water-holding containers infested with larvae) and the morbidity rate are practical community assessments that are low cost, convenient, and improve dengue prevention [1, 14]. These methods were generally used to evaluate dengue prevention outcomes [1, 15].

Although, there was a little evidence confirmed that association between larval indices and dengue infection, some study was positive correlation[16]. In Thailand, the larval indices survey had been the most popular vector surveillance method. The Thai Ministry of Public Health (MOPH) proposed the three larval indices values for estimating dengue risk outbreak which these values showed HI<10, BI<50, and CI<1 [17]. In this study, the survey activities were conducted by village health volunteers (VHVs) working groups, in coordination with primary care units (PCUs) [13, 18]. The activities involved routine practices such as larval surveys, the destruction of mosquito breeding sources, and dengue death prevention campaigns (when budgeting allowed). Larval indices detection and survey activities were not sustainable in these areas because of the brief period of time for surveys and the lack of community participation [19]. VHVs were required to take a corrected training for larval index surveying because they failed to calculate the index levels and occasionally failed to understand the meaning of their surveys. Moreover, health providers did not show the larval index levels to all VHVs or people in the community [13, 18].

The Lansaka district, which encompasses of 11,427 households, and 44 villages across 5 sub-districts in Nakhon Si Thammarat Province. This area was identified as a high-dengue risk area because there were high dengue morbidity rates than standard rate 50 cases/100,000 peoples and mortality rate than 0.2 case per dengue cases[17]. They showed morbidity rate in 2009, 2010, 2011, 2012, and 2013, at 209.1, 833.9, 52.4, 209.8, and 467.9 cases/100,000 peoples, respectively. The dengue mortality rate in 2010 and 2012 were 1.15% (four cases death per 347 dengue infection cases) and 1.23% (one case per 89 dengue cases) [20]. The research team was meeting with 30 representatives from all involving dengue solution in the district showed that many barriers to dengue prevention and control e.g., collecting larval indices survey based on several record formats in each, delay reporting, miss communicating and few using the utilities of larval indices. These obstacle factors were associated with the results from situation assessment of a district which lack of the larval indices data collecting, using, and monitoring [21]. Moreover, there did not present the system or steps to cover all the households, villages, sub-district, and district. Thus, the current study aimed to develop and use a larval indices surveillance system based on appropriate survey methods with community participation from the household to district levels.

## Material and methods

The study design was applied of the community participation action research (CPAR) approach and integrated four concepts[13, 22]: 1) community capacity building, 2) epidemiology, 3) research design for health development, and 4) an online computer program. **Setting:** This study was conducted in Lansaka district, Nakhon Si Thammarat Province, Thailand covering 44 villages across five sub-districts. The Thai Research Fund supported this study (RDG56A0031) from September, 2013 to March, 2015. Approval from the Institute Review Board (IRB) and the Ethical Review Committee for Research Subjects was received from the Health Science Group of Walailak University, Thailand (protocol number 13/047).

### Method

The CPAR approach was divided into five phases: 1) preparation all stakeholders in district, 2) situation assessment, 3) development of the surveillance system, 4) program implementation, and 5) evaluation.

The 1^st^ phase was community preparation. The research team mobilized all of the stakeholders in the district; these stakeholders consisted of 30 representatives from the district health office, a district hospital, nine PCUs, local administrative organizations (LAOs) and community leaders in nine villages, and VHVs. The researcher provided the study objectives to the community leader, and collected consent forms from all participants for data collection and the inclusion of the larval indices survey in household. The objectives, methods, measurements, and utility of the study were described to the stakeholders as part of this step.

The 2^nd^ phase was situation assessment. Assessments included dengue knowledge and understanding of larval indices among VHVs as well as group leaders of the nine villages, interviews of senior VHVs, and a household environment survey. The assessment data were then used for developing and using the larval indices surveillance system.

The 3^rd^ phase was the development of the surveillance system based on community participation and context. This phase was focused on the nine PCUs and a district hospital as ten primary care surveillance centers (PCSCs) in the districts. The activities included 1) the presentation of the situation assessment results, 2) meeting with community leaders and VHVs for dengue and larval indices training, 3) the grouping of VHVs to cover all of the areas in the village, 4) the presentation of the larval indices management model manual, the dengue training book for the larval indices survey, and 5) the development of a computational program for the larval indices online calculation.

The 4^th^ phase was composed of seven steps at the household level and four steps at the district level. The steps at the household level were based on VHVs, PCSCs and the community context, whereas the surveillance steps at the district level were 1) the production of source data groups, 2) district surveillance center (DSC), 3) user data and larval indices information in sub-district groups, and 4) practical guidelines for dengue problem solving.

The 5^th^ phase involved the evaluation of the process and program outcomes. The research team and community participants monitored the system once a month. The resulting data were used as indicators to prevent a dengue outbreak in each village. The data for the surveillance system were collected for four months.

### Questionnaires before and after the intervention

The questionnaires administered before and after the intervention collected information about participants’ basic knowledge of dengue and were developed and tested by the researcher. The format of the self-report consisted of two parts (Part I: General characteristics and Part II: Basic knowledge). Three experts in dengue prevention and control validated of the questionnaire as the contents validity index (CVI) showed 0.89, and reliability was confirmed by a Cronbach’s alpha coefficient of 0.83[23]. The survey took fifteen minutes to complete and consisted of 15 items related to dengue knowledge with three possible answers: yes, no, and unknown. All questions concerned the cause of dengue, the major signs of dengue, the dangers of dengue, mosquito-bite prevention, the mosquito life cycle, and methods of mosquito elimination.

Understanding of the larval indices survey was assessed via 15 open-ended items consisting of four sections: 1) personal information (i.e., sex, education level, experience, and time as a VHV); 2) understanding of the VHV’s role with regard to meaning, type, and calculation (9 items); 3) activities of larval surveillance (2 items), and 4) Problem, barriers, and suggestion to the larval indices survey (4 items).

The computational program is available at http://lim.wu.ac.th. The program was developed for analysis and includes the larval indices from all 44 villages. Larval indices were recorded and analyzed for possible mosquito breeding sites (e.g., drinking water, used water containers, water containers in the bathroom and toilet, cupboard saucers in the kitchen, vases, plant-related containers, and discarded containers surrounding the household).

### Data analysis

The data analysis was performed using the computer program at http://lim.wu.ac.th. Larval indices were analyzed as the ratio of the House Index (HI); the Container Index (CI); and the Breteau Index (BI). An assessment of the larval indices surveillance system was performed using the results from the survey. The types of containers were computed with frequencies and percentages. Pre-and post-intervention tests were performed based on dengue knowledge and the understanding of larval indices with descriptive statistics. The percentages of correct answer were analysis with a chi-squared (χ^2^) test or Fishers' exact test or Chi-square with Yates continuity correction. The chi-square test was done to identify associations of dependent variables (pre-posttest). The statistical analyses p-value less than 0.05 were considered to indicate significance. The qualitative data from the developed and used system were summarized as the model or diagram like steps of process [22, 24].

## Results

### The larval Indices surveillance system of household level

**Fig 1:**
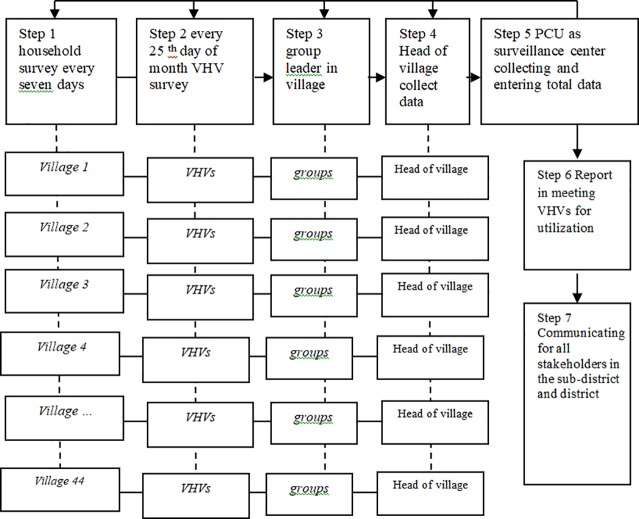
The seven steps of the larval indices surveillance system at the household level.

The surveillance system of households in each sub-district, and consisted of seven steps: First, the householder inspected seven types of water containers in and out household every seven days such as “drinking water containers”, “water containers in bathroom and toilet”, “using water container”, “cupboard sauces”, “vases”, “plant-related containers”, and “discarded containers surrounding the household”. Second, the VHV surveyed the larval indices in 10–15 households to conduct larval indices surveys every 25^th^ day using the “violet book” and sent the larval indices data to the group leader. Third, the group leader collected all of the data from the VHVs in the “blue book”. Fourth, the head of village in each village collected all of the data from the group leaders in the “yellow book”. Fifth, the PCSCs (nine PCU, and a district hospital) collected and recorded the data from all villages using the online program http://Lim.wu.ac.th., analyzed the data and reported the results on the 30^th^ day of every month. Sixth, the larval indices levels of the BI, HI, and CI were reported at the VHV meeting every month. The health workers then proposed the level of larval indices based on information obtained from all VHVs to prevent dengue in high-risk village areas. Seventh, information was communicated by the VHVs to all stakeholders in the community: the sub-district local government organization (SLGO), elementary school, and households. All steps and three record books are shown in Fig 1.

### The larval indices surveillance system of district level

**Fig 2:**
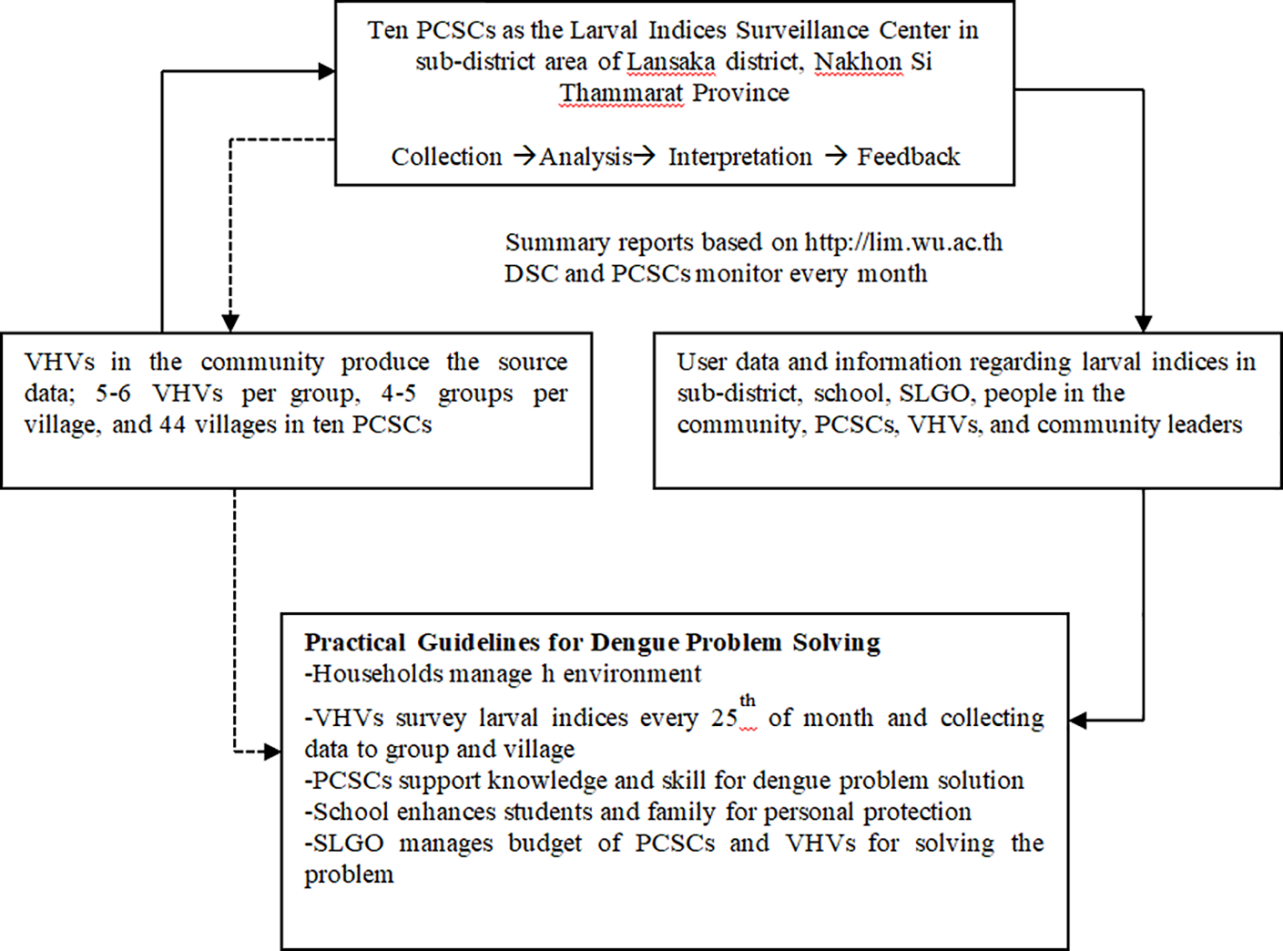
The four components of the larval indices surveillance system at the district level.

The surveillance system of district level represented the overall connection of districts (ten PCSCs). The pattern consisted of 4 components: 1) the production of source data groups, by who were VHVs covering households; 2) DSC were district health center’s officers who monitoring program and system of collecting larval survey data; 3) the user data and information on larval indices in the sub-district and district communities, and 4) the practical guidelines for dengue problem solving in each sub-district (Fig 2).

### VHV dengue knowledge

In comparing the before (n = 503) and after (n = 492) results, the used model showed that almost half of the VHVs were female (46.6% and 45.8%, respectively) with a junior high school education (11.1% and 8.7%, respectively); some VHVs had experience with dengue illness (10.8% and 7.4%, respectively), and had received dengue information (48.7% and 88.8%, respectively). These characteristics did not significantly differ according to the statistical analysis; however, the amount of dengue information increased after the intervention. (no present table)

Dengue knowledge among VHVs significantly increased (*p* < .01), especially with regard to items 1, 2, 4 and 13. The remaining questions showed did not significantly differ (i.e., items 3, 5, 6, 7, 8, 9, 10, 11, 12, 14, and 15; Table 1).

**Table 1:** VHV dengue knowledge, as indicated by the number of correct answers, before and after using the model.

Dengue knowledge	Correct answer (n(%))		χ^2^	*p-value*
	Before (n = 503)	After (n = 492)		
1. *Aedes aegypti* is a conductor of dengue fever	488(97.0)	492(100)	14.897	0.000***
2. All populations in the community are at high risk of dengue fever	485(96.4)	488(99.2)	8.798	0.001**
3. *Aedes aegypti* can fly between houses 50–100 meters away	491(97.6)	486(98.8)	1.904	0.234^*NS*^
4. A very high and sustained fever of 2–7 days is usually a sign of dengue fever	488(97.0)	489(99.4)	7.88	0.007**
5. Dengue fever usually results in a red face and skin bleeding (arm and leg) after a fever for 2–3 days	466(92.6)	449(91.3)	0.644	0.484^*NS*^
6. Dengue treatment must follow only the signs and symptoms because no specific drug exists	466(92.6)	469(95.3)	3.753	0.084^*NS*^
7. Patients with dengue fever can die	494(98.3)	486(98.8)	0.544	0.605^*NS*^
8. *Aedes aegypti* habitually bite during the daytime	489(97.2)	485(98.6)	2.228	0.185^*NS*^
9. *Aedes aegypti* breed in water containers that are clean, such as those in the bathroom and water jars	496(98.6)	490(99.6)	2.693	0.178^*NS*^
10. Coconut shells, broken water jars, and garbage with stagnant water surrounding the household are *Aedes aegypti* breeding sources	502(99.8)	492(100)	0.978	1.000^*NS*^
11. Closed water jars and water containers are a way to prevent mosquito breeding	491(97.6)	481(97.6)	0.025	1.000^*NS*^
12. Eliminate mosquito breeding sources with using clean Containers and change the water every 7 days	501(99.6)	492(100)	1.963	0.500^*NS*^
13. Dry red lime can be placed in a water container to decrease mosquito breeding	451(89.7)	477(97.0)	21.042	0.000***
14. Sleep with a net to prevent mosquito bites	503(100)	491(99.8)	1.023	0.494^*NS*^
15. Citronella is an herb for repelling mosquitoes	500(99.4)	489(99.4)	0.001	1.000^*NS*^

Chi-squared test (χ^2^) Statistic

^NS^ non-significant

* p < .05

**p < .01

***p < .001

### VHV understanding of the larval indices surveillance model

The numbers of VHVs who completed questionnaires before and after the using model were 578 and 487, respectively. Some cases were losing answer in the post-test because of some VHV did not completed the questionnaires. The compared with knowledge of larval indices (items 1–8) before intervention, knowledge after the intervention was significantly increased (*p* < .001), whereas scores on the questions relating to an understanding of larval indices management (items 9, 10, and 11) decreased and significantly differed (*p* < .001, .01, and .05, respectively) (Table 2).

**Table 2:** VHV understanding of the larval indices surveillance system before and after using the model.

Understanding of larval indices	Number (percentage) of correct answers (n (%))		χ^2^	*p-*value
	Before (n = 578)	After (n = 487)		
1. Identify the important larval indices for dengue prevention	462(80.1)	396(81.3)	0.262	0.641^*NS*^
2. Identify the significant types of larval indices	257(40.0)	385(60.0)	132.092	0.000***
3. Descriptive meaning of BI	80(13.8)	332(68.2)	328.929	0.000***
4. Descriptive meaning of HI	16(2.8)	330(6.8)	509.03	0.000***
5. Descriptive meaning of CI	14(2.4)	326(66.9)	506.239	0.000***
6. Identify the result of calculating BI	115(19.9)	153(31.4)	18.6628	0.000***
7. Identify the result of calculating HI	159(27.5)	266(54.6)	81.011	0.000***
8. Identify the result of calculating CI	154(26.6)	207(42.5)	29.677	0.000***
9. Conducting prevention activities when larval breeding is found	519(89.8)	445(91.4)	0.772	0.402^*NS*^
10. PCU reported larval indices to all stakeholders in community	27(4.7)	347(71.3)	514.234	0.000***
11. Utility of using larval indices survey	420(72.7)	380(78.0)	4.069	0.046*

Chi-squared test (χ^2^) Statistic

^NS^ non-significant

* p < .05

**p < .01

***p < .001

The report showed a decrease in the percentage before and after intervention with regard to the questions item number 1) “Have problems using the larval survey” (89.3% and 81.7%, respectively), 2) “Have barriers with regard to identifying and calculating larval indices” (83.2% and 80.7%, respectively), 3) “Needs support with the larval indices survey” (59.9% and 49.5%, respectively), and 4) “Have suggestion with larval indices surveillance system”“ (42.4% and 37.6%, respectively). For the VHVs, the comparison between before and after the intervention did not reveal a change; only two significant decrease was observed (“have barriers with regard to identifying and calculating larval indices”, and “Need support with the larval indices survey”) *p* < .001, (Table 3).

**Table 3:** VHV understanding of larval indices with problems, barriers, and needs before and after developing the model.

Understanding of larval indices	Number (percentage) of question (n (%))		χ^2^	*p-value*
	Before (n = 578)	After (n = 487)		
1. Have problems with larval indices survey	516(89.3)	398(81.7)	11.764	0.001**
2. Have barriers to identifying and calculating larval indices	481(83.2)	393(80.7)	0.975	0.298 ^*NS*^
3. Need support with the larval indices survey	346(59.9)	241(49.5)	11.085	0.001**
4. Have suggestion with larval indices surveillance system	245(42.4)	183(37.6)	2.348	0.117^*NS*^

Chi-squared test (χ^2^) Statistic

^NS^ non-significant

* p < .05

**p < .01

***p < .001

### Larval indices

**Fig 3:**
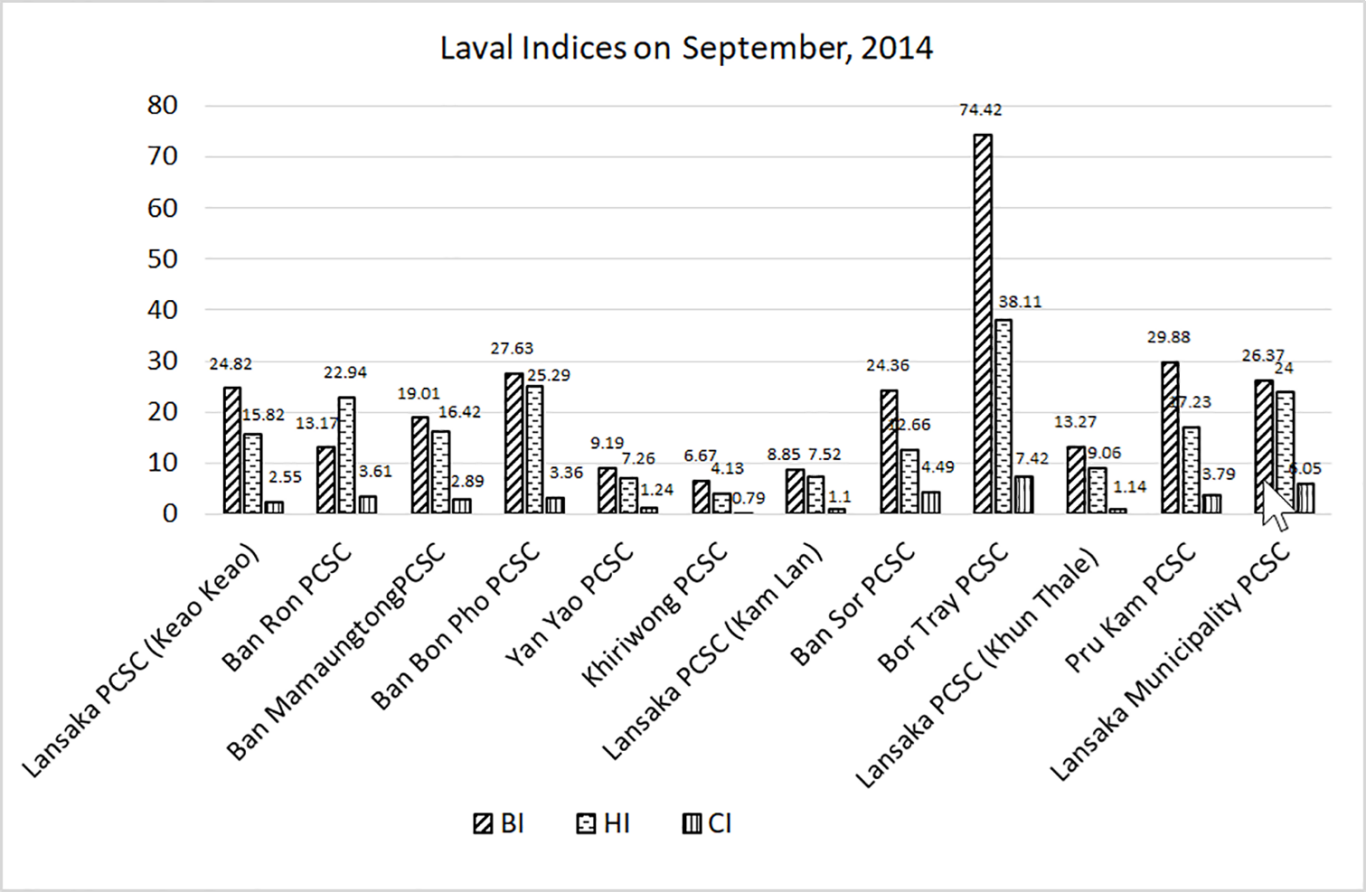
Larval indices level of ten PCSCs (nine PCSCs and Lansaka PCSC devised into three areas from different sub-districts) on September, 2014 (After conducting study 12 months).

The results showed the output of the surveillance system study that followed the seven steps. The larval indices levels of the 44 villages in ten PCSCs such as: 1) Ban Bon Pho PCSC (3 villages); 2) Yan Yao PCSC (6 villages); 3) Khiriwong PCSC (4 villages); 4) Ban Sor PCSC (5 villages); 5) Bor Tray PCSC (5 villages); 6) Pru Kam PCSC (7 villages); 7) Ban Mamaungtong PCSC (4 villages); 8) Ban Ron PCSC (4 villages); 9) Lansaka PCSC (6 villages, equally 2 villages in Keao Keao, Kam Lon, and Khun Thale sub-districts) and 10) Lansaka Municipality PCSC (a Municipality) were reposted via the program found at http://lim.wu.ac.th. For example, the larval indices report of September, 2514 (12 months after conducting this study) found that almost all PCSCs showed that the monthly monitoring process was able to lower the larval indices level closer to the standard level established by the Thai MOPH (Fig 3).

### Dengue mortality rate

There shows an area at high risk for dengue, and it had high morbidity rates of 209.1, 833.9, 52.4, 209.8, and 467.9 cases/100,000 people in 2009, 2010, 2011, 2012, and 2013, respectively. In 2014, the current study was launched to seek sustainable solutions to the dengue problem in the area based on the participation of all stakeholders. The model involved the participation of all areas or groups, and a surveillance system of mosquito breeding sources in households (larval indices) collected by VHVs was provided to the group leaders of each village and then to PCSCs to enable the use of the computer program at http://lim.wu.ac.th. Feedback on the data was then provided to stakeholders to organize dengue activities. The morbidity rate was decreased from 164.9 cases/100,000 people in year 2014 to 151 cases/100,000 people in year 2015.

## Discussion

The larval indices surveillance system, the model process was divided into two levels; the household to sub-district level, and the district level included five sub-districts. The model was designed and used based on participatory of all stakeholders such as the community hospital, eight PCUs, the district-chief officers, public health officials, community leaders, and five LAOs in Lansaka district. The surveillance system consisted of one DSC and ten PCSCs. The system was adapted to the context of the rural community and community participation, integrating the concept of CPAR as a new approach [22]. In each month, a PCSC was conducting and monitoring the seven steps of the household level (seven steps) to district level. The system was implemented via all of the stakeholders at the household, village, sub-district, and district levels who engaged participants in solving the dengue problem associated the prevention and control guideline [25]. Moreover, the steps of the new approaches of larval indices surveillance were reported in association with the dengue prevention strategy of the World Health Organization (WHO), because of its was the proactive surveillance system, the computer program and the capacity-building community [25, 26].

VHV’s dengue knowledge and the larval indices surveillance system is an important and active strategy for dengue problem solving. Using a community-based approach, as indicated by the “before” intervention data, VHVs performed well with regard to dengue knowledge (15 items), but their knowledge of larval indices (15 items) and calculation skills (15 items) was poor [3, 27, 28]. The dengue knowledge of the VHVs was acceptable, but their understanding of the larval indices was poor. Thus, an education program required to integrate the larval indices management system and the effectiveness of the vector control program [29, 30]. The comparisons with the “after” intervention scores showed an increase in knowledge of both dengue and larval indices. These results suggest that participatory intervention increases the knowledge capacity of community members. The larval indices level and dengue morbidity rate after the intervention were lower than those before the intervention. These results suggest that an active larval indices surveillance system improves awareness among all stakeholders.

Larval Indices and Mortality Rate from study found the larval indices level relating with the decreasing trend of dengue morbidity rate after using the model. It confirmed some report which association between larval indices and dengue transmission[16, 31]. The practical work conducted in the Thai public health system includes PCUs as well as VHVs working toward dengue prevention and control in communities; these aspects have been the subject of previous studies in southern Thailand [13, 18] and other areas [19]. The groups of leaders were crucial in the surveys of mosquito breeding, the communication of larval indices, and the implementation of dengue prevention activities in households and villages. However, the study needs proving the relationship of larval indices and dengue transmission, factor involving, and continuous monitoring the using the model because the sustained success of a dengue prevention program requires a long-term observation period (i.e., 3 to 7 years) [19, 32].

## Conclusions

The “Lansaka Model” is the name of the dengue solution program in Lansaka district, southern Thailand. This model larval indices surveillance system was interconnected, covering 11,427 households, 44 villages, five sub-districts, a municipality and the entire district. The steps of the study, including community participation, situation analysis, planning, development, and evaluation of the surveillance system, is described above. The test results from before the intervention clearly show basic dengue knowledge among the VHVs; however, these workers had a poor understanding of the larval indices. Almost all of the VHVs of the ten PCSCs (PCUs/a district hospital/a municipality) showed an increased understanding of larval indices after the intervention. The active surveillance consisted of seven steps in household level, and four components for monitoring in district level. Moreover, the VHVs required the officials of all surveillance centers to coordinate, teach, coach, and conduct the dengue project in their villages. The new surveillance system resulted in decreased larval indices levels as well as and a decreased morbidity rate. However, the reported levels remained higher than the Thai MOPH standard. Thus, all stakeholders should conduct continuous monitoring to achieve a sustainable dengue solution.

## Limitation

The study cannot control the same participants in pre-posttest because the community is dynamic status. The study was conducting on community participatory action research (CPAR). It focused on develop the appropriated technique for dengue problem solving. However, the VHVs’ characteristics did not differ in pre and posttest. Then, the study needs proving the relationship of larval indices and dengue transmission, factor involving, and continuous monitoring the using the model.
